# Nephrotic syndrome with a nephritic component associated with toxoplasmosis in an immunocompetent young man.

**Published:** 2012-09-30

**Authors:** Julio E Barrios, Claudia Duran Botello, Tania González Velásquez

**Affiliations:** a Head of the Pediatric Nephrology Team, Napoleón Franco Pareja Hospital, Cartagena, Colombia. E-mail: taniagonzalezvelasquez@gmail.com; b Department of Pediatrics, Faculty of Medicine, University of Cartagena Colombia. E-mail: ejuliob@gmail.com; c School of Medicine, University of Cartagena, Colombia.

**Keywords:** Proteinuria, renal disease, *Toxoplasma gondii*, immunocompetent, mixed nephrotic syndrome

## Abstract

**Introduction:**

Although the association of infection by toxoplasmosis with the development of nephrotic syndrome is uncommon, cases of this association have nevertheless been reported in the literature for more than two decades, not only for congenital toxoplasmosis, but also in acquired cases, and occasionally in immunocompetent patients.

**Development:**

A case is presented of an immunocompetent patient aged 15 with clinical and laboratory indications of nephrotic/nephritic syndrome, in whom serological tests showed *Toxoplasma* infection.

**Conclusion:**

The presentation of nephrotic syndrome in ages where it is not commonly seen, leads to clinical suspicion of secondary causes. Active search for possible causes should include common tropical infections.

## Abstract (es)

**Introducción:**

Aunque no es frecuente la asociación de infección por toxoplasmosis y el desarrollo de síndrome nefrótico, desde hace más de dos décadas se han reportados casos de esta asociación en la literatura médica, no solo en casos de toxoplasmosis congénita, sino también adquirida, y esporádicamente en inmunocompetentes.

**Desarrollo:**

Se presenta un caso de un paciente de 15 años inmunocompetente con signos clínicos y paraclínicos de síndrome nefrótico/nefrítico, en quien se determinó por pruebas serológica infección aguda por *Toxoplasma*.

**Conclusión:**

La presentación del Síndrome nefrótico en edades poco usuales, induce a la sospecha clínica de causas secundaria, la búsqueda activa de las posibles causas debe incluir infecciones parasitaria.

## Introduction

Nephrotic syndrome presents clinically with massive proteinuria and hypoalbuminemia, accompanied by variable forms of edema, hyperlipidemia, and lipiduria, all as a result of increased glomerular permeability^1^, and it can be associated with nephritic syndrome when some or all of its clinical concomitant manifestations (arterial hypertension, hematuria, hypocomplementemia and renal failure) are present. In these cases it is recognized as mixed or atypical nephrotic syndrome and comprises signs of nephritic and nephritic syndrome, this usually occurring when the glomerular lesion is principally in the mesangium and the membrane^2^. 

With regard to its etiology, nephrotic syndrome is recognized as idiopathic and secondary; idiopathic SN appears as a frequent pathology in pediatrics registering approximately 16 in 100,000 children under 18 years old^3^, approximately 50% of children affected are between the ages of 1 and 4 years, and 75% are less than 10 years of age^4^. The presentation outside of this age group requires the clinician to look for secondary causes of the etiology, including infectious causes such as parasites. 

Glomerulopathies associated with parasites are initiated by the depression of the mesangium of immune complexes which contain parasite antigens, therefore this damage is more pronounced with parasites which live in the bloodstream such as *Plasmodium* and *Schistosoma* ^5^. In the case of *Toxoplasma gondii*, although by definition it is an intracellular parasite, there also occurs glomerular damage caused by immune complex which contains a toxoplasmosis antigen identified in infected mice^6^, and in congenital nephrotic syndrome due to toxoplasmosis^7^. 

Toxoplasmosis is a complex disease which usually follows an asymptomatic course. The involvement of organs and systems usually presents in immunocompromised patients and in children infected via the transplacental route. However, in the past decade acute cases of the disease in immunocompetent patients have been described. For serological diagnosis, it is noted that the first antibodies which appear are IgM antibodies followed by IgG antibodies and the former disappear more quickly than the latter. The discovery of anti-*Toxoplasma gondii *IgM or IgG antibodies in a serum sample is limited to establishing that the host has been infected at some point in the past. The degree of vitality of the IgG establishes how temporary is their character, the low vitality indicates a recent infection - fewer than 6 months previously. The presence of IgG antibodies of low vigor is not a reliable indication of acute infection. Therefore the suggested method to establish acute infection is to collect two samples from the same individual, the second collected 2-4 weeks after the first. An increase in the antibody level in the second sample indicates an acute infection^8^. 

The aim of describing this case is to describe the possible etiological role of *Toxoplasma* infection as a cause of mixed nephrotic syndrome. 

## Clinical case

A 15 year old male patient was admitted to the emergency room, previously healthy and not taking any medication. He presented symptoms of 5 days of edema initially only in the eyelids which later progressed to the lower limbs, associated in the previous 24 hours with severe abdominal pain and fever spikes. 

Physical examination showed him to be cold. He weight was 55 kg, dry weight: 47 kg (percentile 25), height: 1.56 m , 1.60 cm (percentile 5), heart rate: 88/min, respirations: 18/min, blood pressure: 160/90 (systolic blood pressure: > percentile 99 and diastolic blood pressure: percentile 99), temperature: 36.4 ºC. He had extensive facial edema. There were symmetric rhythmic heart sounds without murmurs, breath sounds with scattered rhonchi. The abdomen was soft, painful on palpation, with no epigastric visceromegaly. The lower limb extremities showed mild grade II edema, bilateral inguinal lymphadenopathy of 5 cm, painful on palpation. There was no neurological deficit. 

Initial laboratory tests showed anemia, thrombocytopenia, leukocytosis with mild eosinophilia, urinalysis showed proteinuria and hematuria (Table 1). Renal function tests showed altered kidney function and random proteinuria and creatinuria, and later proteinuria at 24 hours, positive parameters for nephrotic syndrome. Lipid profile was carried out as well as serum protein tests. Infection and rheumatological screening showed a negative rheumatoid profile and positive serology for *Toxoplasma* (Table 1). 

X-ray results Abdominal ultrasound showed free fluid in the bilateral costophrenic spaces. Liver morphology and size were normal. There was free fluid in the abdominal cavity. Kidney morphology, size and localization were normal. There was widespread increase in ecogenicity. Chest X-ray showed obscuring of the bilateral costophrenic recesses. Echocardiogram showed mild left ventricular dilation with no hemodynamic repercussions or pericardial effusion. 

The patient was initially treated with furosemide 1 mg/kg/dose with albendazole 400 mg daily for three days. After 24 hours of treatment a negative balance was achieved. The patient remained hypertensive (150/80 mmHg) and amlodipine 10 mg daily was added, which reduced blood pressure to 140/90 mmHg. In association with the antihypertensive hydrochlorothiazide 50 mg daily, a blood pressure at percentile 90 was achieved (120/70). For deparasitation, prednisolone 40 mg SI daily was started plus omeprazole (20 mg daily) and calcium carbonate plus vitamin D (600 mg daily). The patient had a slow clinical course, the edema persisting. Fluid was restricted to 1500 mL SI daily and a negative fluid balance. 

Following a positive diagnosis of acute toxoplasmosis, treatment was initiated with pyrimethamine and sulfadiazine (50 mg/1.5g daily) plus folic acid (1 mg daily), and biweekly blood counts to check for possible toxic medullary effects. A few days after starting treatment, an improvement was achieved both clinically and according to laboratory test results. 

Renal biopsy was performed, which showed glomerulonephritis of membranoproliferative pattern, associated with immune complex deposits. 

Following six weeks of treatment with Pyrimethamine, sulfadiazine and prednisolone, 40 mg daily, the patient's tests showed signs of renal failure stage III (Table 2) . He was continued with prednisolone, 40 mg SI, alternate days and a monthly pulse of Cyclophosmide. 

**Table 1:**
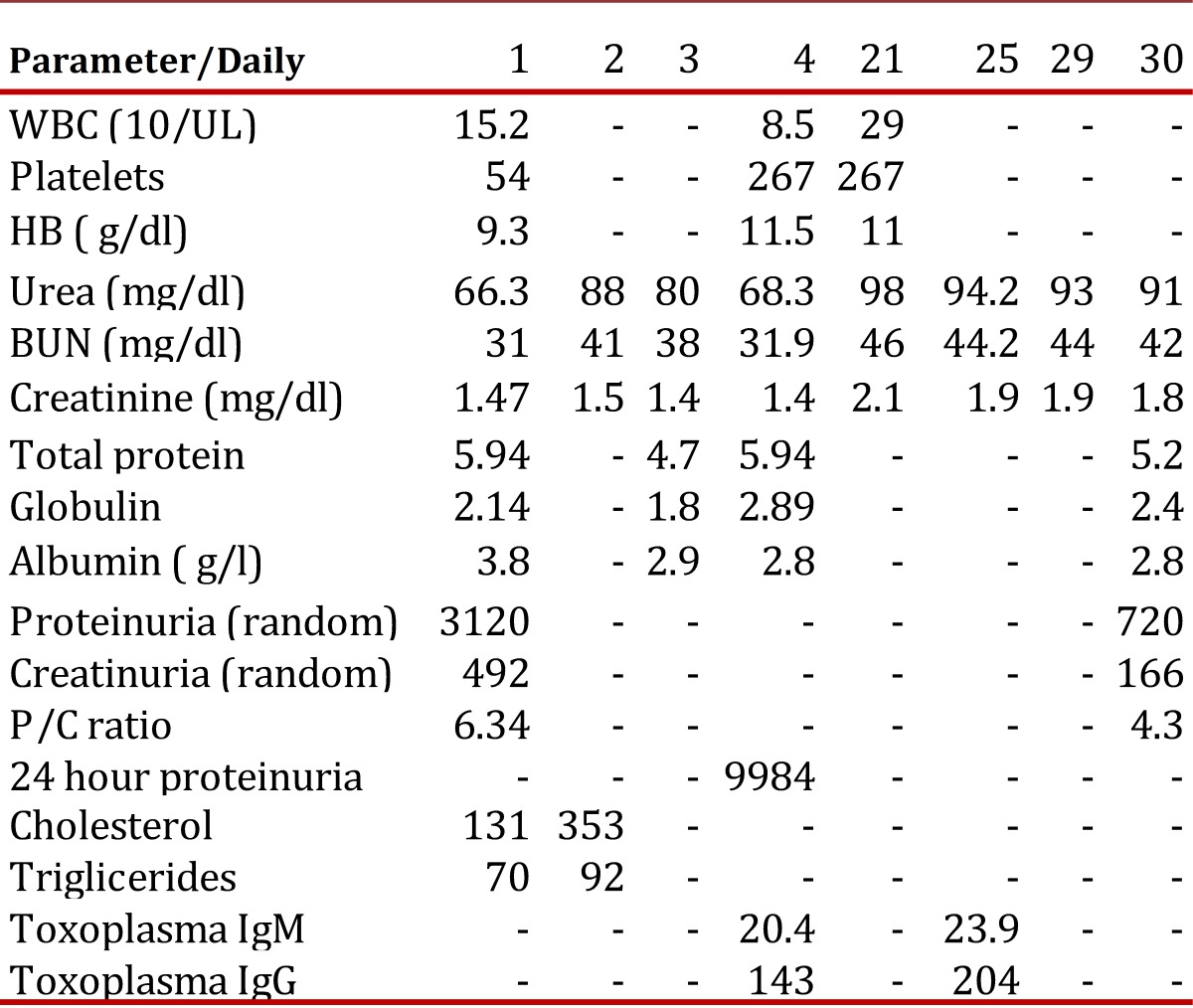
Analysis on admission and during hospital stay

**Table 2:**
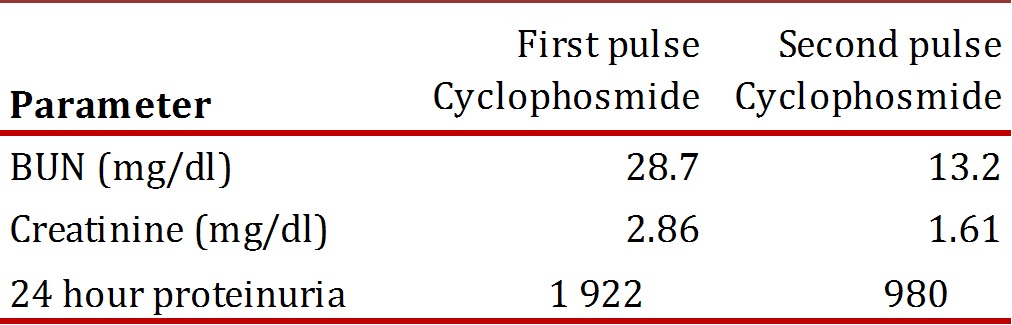
Analysis the before first pulse cyclophosmide and second pulse cyclophosmide. BUN:blood urea nitrogen

## Discussion

The initial clinical picture presented as nephritic syndrome, which in its clinical course is associated with clinical and laboratory signs of nephrotic syndrome. The result is mixed nephrotic syndrome. 

Given the unusual presentation of idiopathic nephrotic syndrome in patients older than 10 years old as well as the peculiar clinical evolution of nephritic syndrome to mixed nephrotic syndrome, studies of possible etiologies were carried out. Although there was no clinical likelihood of toxoplasmosis, the discovery of anti-toxoplasma antibodies type IgM and IgG nevertheless suggests infection as a possible cause. 

The suspected diagnosis of acute toxoplasmosis was raised by the positive serology, both IgG and IgM specific; confirmation was obtained on demonstrating a significant rise in IgG specific antibody titer from 1/118 to 1/240 and IgM of 1/20.38 to 1/23.82. The findings of IgM reagent in the first analysis was not sufficient to diagnose acute toxoplasmosis given that IgM antibodies can persist up to 1 year or more after primary infection^9^. 

Although it is unknown why an apparently immunocompetent patient such as this young man develops acute toxoplasmosis with relevant clinical signs requiring specific management, there are descriptions in the literature of such atypical presentations of the infection in immunocompetent patients^10^ ^,^ ^11^. 

The case of this atypical nephrotic syndrome caused by *Toxoplasma* infection, producing clinical signs of the syndrome due to possible immunological reaction is also unusual in terms of its cause, the age of the patient, the immunocompetent status of the patient as well as the mixed clinical picture. 

## Conclusion

In identifying etiologies of atypical nephritic syndrome presenting in uncommon pediatric age groups, an active search for infections, including parasites, must be undertaken. 

In Colombia infection by *Toxoplasma* is common. It is considered that half of the population has had contact with the parasite, therefore, positive serology for this infection and the diagnosis of mixed nephrotic syndrome should make us think in toxoplasmosis as one of the possible etiologies of this complex entity. 

Studies and description of cases like this one are required, with an outline of serological data of acute infection as well as a time correlation to the mixed nephrotic syndrome, defining the prevalence of this association and possible etiologic contribution to the causes of renal severe acute as that of our patient. 
